# Using an Administrative and Clinical Database to Determine the Early Spread of COVID-19 at the US Department of Veterans Affairs during the Beginning of the 2019–2020 Flu Season: A Retrospective Longitudinal Study

**DOI:** 10.3390/v14020200

**Published:** 2022-01-20

**Authors:** Lilia R. Lukowsky, Claudia Der-Martirosian, William Neil Steers, Kiran S. Kamble, Aram Dobalian

**Affiliations:** 1 Veteran Emergency Management Evaluation Center (VEMEC), US Department of Veterans Affairs, North Hills, CA 91343, USA; Claudia.Der-Martirosian@va.gov (C.D.-M.); William.Steers@va.gov (W.N.S.); Aram.Dobalian@va.gov (A.D.); 2 Division of General Internal Medicine and Health Services Research, David Geffen School of Medicine, University of California, Los Angeles, CA 90095, USA; 3 School of Public Health, University of Memphis, Memphis, TN 38152, USA; Kiran.Kamble@memphis.edu

**Keywords:** COVID-19 symptoms, Veterans, influenza-like illnesses

## Abstract

Background. Previous studies examining the early spread of COVID-19 have used influenza-like illnesses (ILIs) to determine the early spread of COVID-19. We used COVID-19 case definition to identify COVID-like symptoms (CLS) independently of other influenza-like illnesses (ILIs). Methods. Using data from Emergency Department (ED) visits at VA Medical Centers in CA, TX, and FL, we compared weekly rates of CLS, ILIs, and non-influenza ILIs encounters during five consecutive flu seasons (2015–2020) and estimated the risk of developing each illness during the first 23 weeks of the 2019–2020 season compared to previous seasons. Results. Patients with CLS were significantly more likely to visit the ED during the first 23 weeks of the 2019–2020 compared to prior seasons, while ED visits for influenza and non-influenza ILIs did not differ substantially. Adjusted CLS risk was significantly lower for all seasons relative to the 2019–2020 season: RR15–16 = 0.72, 0.75, 0.72; RR16–17 = 0.81, 0.77, 0.79; RR17–18 = 0.80, 0.89, 0.83; RR18–19 = 0.82, 0.96, 0.81, in CA, TX, and FL, respectively. Conclusions. The observed increase in ED visits for CLS indicates the likely spread of COVID-19 in the US earlier than previously reported. VA data could potentially help identify emerging infectious diseases and supplement existing syndromic surveillance systems.

## 1. Introduction

Evidence suggests that COVID-19 might have been present in China [1,2], Europe [3,4,5,6,7,8] and in the United States (US) [9,10,11] much earlier than 20 January 2020 when the first COVID-19 case [9], or January 30 when the first person-to-person transmission were reported in the US [12]. In the US, the early spread of COVID-19 was suggested by increases in Influenza-Like Illness (ILI) reported by the ILI Surveillance Network (ILINet), the Centers for Disease Control and Prevention (CDC) database [13,14,15,16]. An online survey in the US showed an increase in ILI among influenza-vaccinated adults in 2019–2020 compared to the previous year [17], and a study using electronic health records reported a significant increase in ED visits for cough and excess hospitalizations for acute respiratory failure between late December 2019 and February 2020 [18].

To our knowledge, no study has examined ILI trends as a tool for monitoring COVID-19 among Veterans receiving care from the VA. Accordingly, we conducted a retrospective, longitudinal study to evaluate pattens for ILI during the 2019–2020 season by comparing its first 23 weeks (October 2019–February 2020) to the previous four seasons. Because patients with COVID-19 often experience symptoms resembling ILI, it is important to differentiate between ED visits for influenza and non-Influenza-Like illnesses and encounters for possible COVID-19 infections. It is especially important as US Veterans tend to be older, have a higher number of comorbidities, lower incomes, and are more likely to be from racial and ethnic minorities compared to the general adult population [19,20] Unlike prior studies, we used the established case definition for COVID-19 [21] in the absence of laboratory tests.

We hypothesized that ED visits for COVID-like symptoms (CLS) in early 2019–2020 would increase before March 2020 when the initial COVID-19 surge triggered stay-at-home orders across the US. We expected to observe increases in the rates of ED visits for these symptoms in the 2019–2020 season, while rates for ED visits related to influenza and non-influenza ILIs would remain unchanged.

## 2. Materials and Methods

We used administrative and clinical data from the VA Corporate Data Warehouse (CDW) from 1 October 2015 to 30 September 2020. Our key independent variable was the season in which ED encounters occurred, starting at the week beginning 1 October (calendar week 40) and ending at the week beginning 30 September the following year (calendar week 39). Three measures of ILI-related ED visits were used as dependent variables in our analyses: CLS, influenza, and non-influenza ILIs [22]. We used ICD-10-CM diagnostic codes to identify influenza and non-influenza ILI. Additionally, we employed the COVID-19 case definition (21) and official ICD-10-CM coding guidelines [23] to identify CLS encounters (CSTE’s clinical criteria for reporting a COVID-19 case require at least two of the following symptoms: fever (R50.9), chills (R68.83), rigors(R50.81), myalgia (M79.10, R52), headache (R51), sore throat (R07.0, J0.29) new olfactory/taste disorders (R43.8, R43.9); or at least one of the following symptoms: cough (R05), shortness of breath/difficulty breathing (R06.00, R06.02, R06.03) or severe respiratory illness with at least one of the following: pneumonia (J12.89) or acute respiratory distress syndrome (ARDS) (J80). We used J09-J11 ICD-10-CM codes to identity influenza diagnoses; and B97.89, H66.90-H66.99, J00, J01.9, J02.9, J06.9, J12.89, J12.9, J18.8, J18.9, J20.9, J40 to identify non-influenza ILI).

We calculated the rates of weekly encounters for CLS, influenza, and non-influenza ILI per 1000 ED encounters to examine the differences in trends among the different seasons, conducting separate analyses for California, Texas, and Florida. These states had the largest numbers of COVID-19 cases outside of New York City (NYC), and therefore, could provide a general sense of the early spread of COVID-19 in the US, unlike the early outbreak in NYC. VA uses centralized guidelines for reporting symptoms and diagnoses using ICD-10-CM, which should minimize differences in reporting among the states and specific sites.

Furthermore, we assessed differences in CLS, influenza, and non-influenza ILI diagnoses for each state between the first 23 weeks of each season, from calendar week 40 (first week of October) to calendar week 10 of the following year (last week of February), the period in 2019–2020 before COVID-19 tests and diagnostic criteria became available. We used two-sample *t*-tests for the mean number of encounters for each category, comparing the early weeks of the 2019–2020 season to each preceding season. We created binary indicators for ED visits for CLS, influenza or non-influenza ILI, and fitted mixed effects logistic regression models for correlated binary outcomes for each indicator separately for CA, TX, and FL. Next, we transformed the season variable coefficients to marginal probabilities for CLS, influenza, and non-influenza ILI, and expressed the seasonal effects as risk ratios (RR) between the first 23 weeks of 2019–2020 and each of the four previous seasons, adjusting for age, gender, race, and marital status. Analyses were performed using SAS Enterprise Guide 7.1 (SAS Institute, Cary, NC, USA) and STATA 16 (StataCorp LLC, College Station, TX, USA). The study was conducted according to the guidelines of the Declaration of Helsinki and approved by the VA Greater Los Angeles Healthcare System Institutional Review Board (Project Number: 1616040. Approval Date: 8 March 2020).

## 3. Results

Compared to the 2019–2020 season, we found that the unadjusted rates for CLS encounters were significantly lower during the first 23 weeks of the previous four seasons (Table 1). Furthermore, we observed a higher, but not significant difference in the rates of influenza during the 2017–2018 season, while for the other seasons, the rates were significantly lower compared to 2019–2020. For non-influenza ILI, we observed lower, but not significant, differences in rates when comparing 2019–2020 to the other seasons.

In adjusted analyses, risk for CLS was significantly lower for all seasons in every state, compared to the first 23 weeks of the 2019–2020 (RR15–16 = 0.72, 0.75, 0.72; RR16–17 = 0.81, 0.77, 0.79; RR17–18 = 0.80, 0.89, 0.83; RR18–19 = 0.82, 0.96, 0.81,) in CA, TX, and FL, respectively. However, the adjusted RR for influenza for the same period for all three states during the 2017–2018 season was significantly higher (RR17–18 = 1.24, 1.56, 1.41) compared to the 2019–2020. For the remaining seasons, influenza risk was significantly lower (RR15–16 = 0.29, 0.15, 0.39; RR16–17 = 0.45, 0.32, 0.43; RR18–19 = 0.34, 0.35, 0.50). Non-influenza ILI showed no clear patterns when the risk for 2019–2020 was compared to previous seasons (RR15–16 = 1.03, 0.93, 0.92; RR16–17 = 1.05, 1.10, 1.07; RR17–18 = 1.08, 1.10, 1.06; RR18–19 = 0.91, 1.01, 0.98).

**Figure 1:**
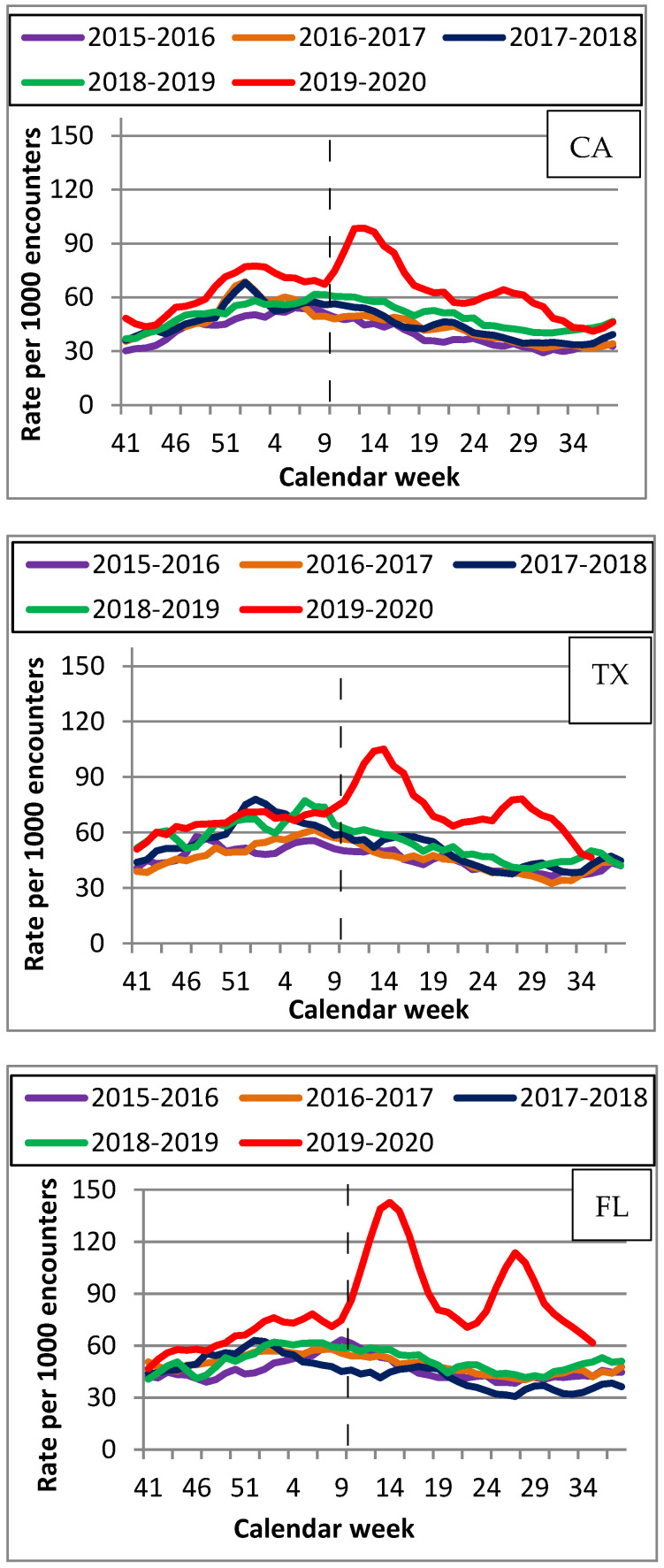
Rates per 1000 ED encounters for COVID-like symptoms by state (CA, TX, FL) for 5 flu seasons (2015–2020).

**Figure 2:**
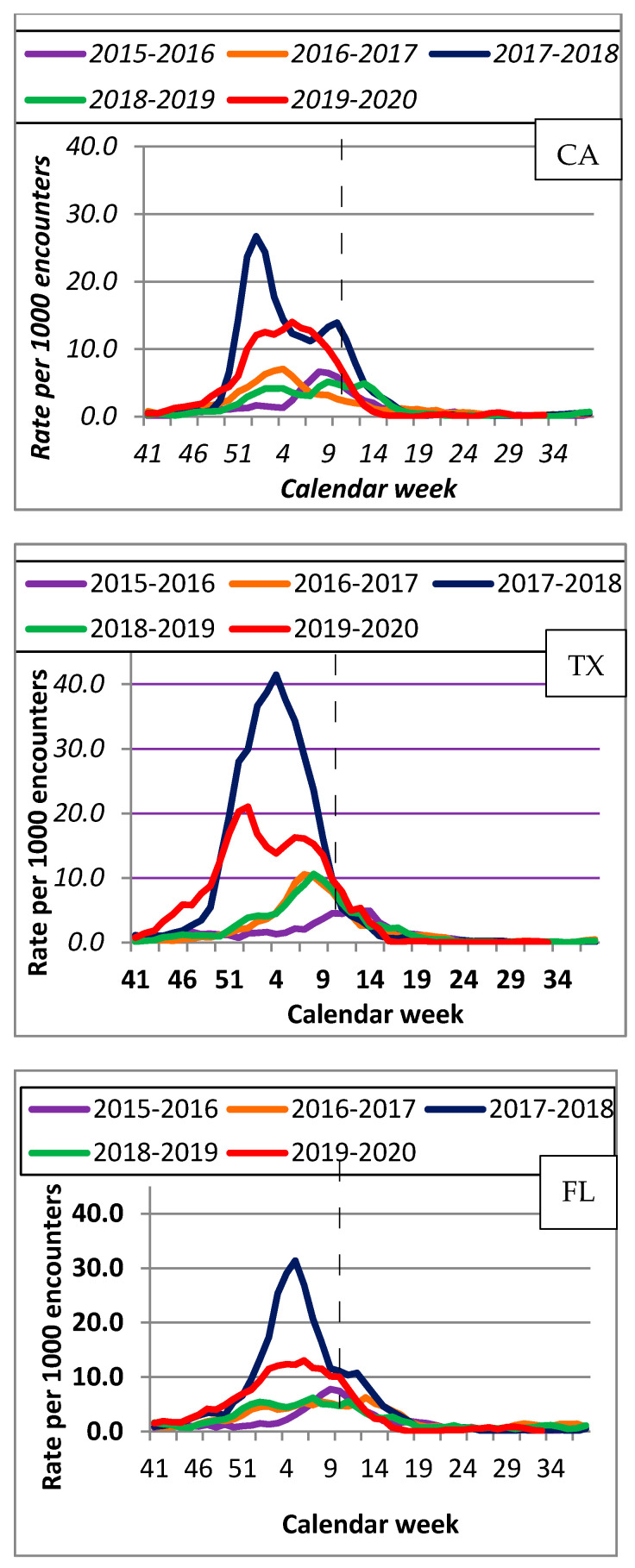
Rates per 1000 ED encounters for influenza diagnoses by state (CA, TX, FL) for 5 flu seasons (2015–2020).

**Figure 3:**
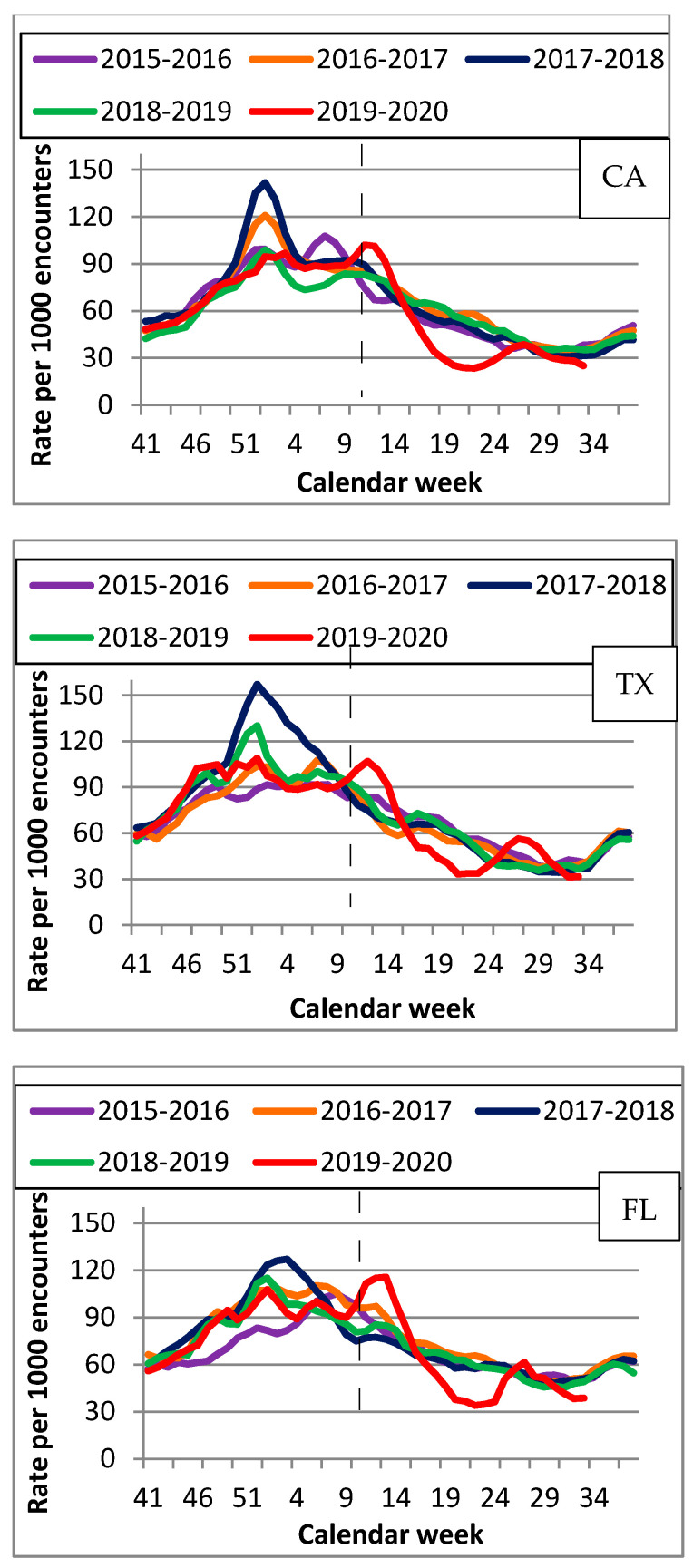
Rates per 1000 ED encounters for non-influenza ILI by state (CA, TX, FL) for 5 flu seasons (2015–2020).

Figure 1, Figure 2 and Figure 3 display the trends in CLS, influenza, and non-influenza ILI for each state showing the weekly rates for each condition per 1000 ED encounters. There was an increase in CLS during the 2019–2020 compared to the other seasons, including during the first 23 weeks (Figure 1). The highest ED encounter rates occurred during calendar week 12 in CA (104/1000), week 13 in TX (112/1000), and week 14 in FL (152/1000).

Figure 2 illustrates patterns for influenza with the highest rates during the 2017–2018 season, followed by 2019–2020. In CA, the highest number of influenza ED encounters occurred during calendar week 1: 32/1000 in the 2017–2018 season and 17/1000 during 2019–2020. In TX, the peak for influenza encounters of 45/1000 in 2017–2018 occurred during week 3, and week 52 in 2019–2020 (24/1000). In FL, it occurred during week 4 with 36/1000 encounters in 2017–2018, and during week 2 with 13/1000 encounters for 2019–2020.

Figure 3 illustrates the ED encounter rates for non-influenza ILI. During 2019–2020, the highest rates were observed much later than during the previous seasons and coincided with CLS rates for the same season (Figure S1a–e), peaking during week 12 with 104/1000 encounters in CA and 130/1000 in FL. In TX, the highest rates of 113/1000 encounters were recorded during week 13. Figure S2a–c indicate ED encounters among patients presenting with shortness of breath, cough, and fever for each state during the same time period. Encounters for shortness of breath and fever peaked during week 14 in CA and TX, and week 15 in FL, and for cough during week 12 in CA and TX and week 13 in FL.

It should be noted that there were no differences in demographic characteristics (age, gender, race, marital status) of the study population between the three states (see Tables S1 and S2).

## 4. Discussion

An increase in the rate of ED encounters for CLS occurred during both March-September 2020, a known period of the initial COVID-19 surge, and October 2019-February 2020, before there was any known spread of COVID-19. While previous studies reported an early presence of COVID-19 due to increases in ILI or non-influenza ILI during early 2019–2020 [13,14,15,16,17,18], our findings offer a more detailed perspective as we examined visits for symptoms consistent with COVID-19 and separately for influenza and non-influenza-ILI. Since all three symptom categories were not mutually exclusive, it can be difficult to evaluate whether the increases observed in other studies with combined categories were indeed due to increases in CLS. We observed that 15–20% of patients with CLS also had an influenza diagnosis, and 20–25% had non-influenza ILI diagnosis during the same ED visit). These percentages were consistent for all seasons in every state, and therefore could not account for the unique CLS patterns observed during the 2019–2020 season. Most of the overlap between CLS, influenza, and non-influenza ILI symptoms occurred early in each season as there was a substantial decrease in non-influenza ILI cases, while cases of influenza were close to zero per week later in each season. VA continued administering influenza tests during the second part of the 2019–2020 season during the COVID-19 surge, indicating that the drop in influenza cases was due to a decrease in influenza activity rather than a lack of testing. Separating CLS from other ILI allowed us to assess symptoms consistent with COVID-19 and highlight changes between seasons for each diagnostic category.

The patterns for influenza during 2019–2020 show an early increase in activity between late December and early January, which is consistent with flu patterns reported in CA, TX, and FL, as well as nationwide among the general population for the same period [24,25,26,27]. Increases early in the season could indicate actual influenza activity. However, they may also partially be due to an increase in CLS because of overlap between CLS and influenza symptoms. The increase in the 2019–2020 season was statistically significant for influenza compared to other seasons, except for 2017–2018, which had the highest rate. These observations suggest that some of the influenza activity early in the 2019–2020 season could be due to an increase in CLS. The rates for ED visits for influenza were consistently about five-fold lower than for CLS. Therefore, it is unlikely that the increase in CLS during the first 23 weeks of 2019–2020 was affected by influenza activity.

Although we did not observe a substantial increase in non-influenza ILI early in the 2019–2020 season, we found a slight increase in early/mid-March 2020 followed by a decrease between weeks 17 and 25, which could most likely be attributed to better COVID-19 diagnostics as well as the implementations of masking and social distancing. (Figure 3 and Figure S1a). This also could be due to CLS because there was an overlap between CLS and non-influenza ILI diagnoses among ED patients. While the patterns were consistent between seasons and states, the time of increase in non-influenza ILI was different in 2019–2020 (Figure 3 and Figure S1a) compared to other seasons and was consistent with COVID-19 patterns. It is unlikely that the observed increase in CLS early in 2019–2020 was due to non-influenza ILI because we did not observe an increase in non-influenza ILI during the first 23 weeks. Additionally, the increase in mid-March observed for both CLS and non-influenza ILI coincides with the nationwide COVID-19 surge. An increase in non-influenza ILI around that time might reflect difficulties with COVID-19 testing, which could potentially have led to non-influenza ILI diagnoses in COVID-19 patients. Additionally, we observed an increase in ED encounters for patients presenting with shortness of breath and cough in all three states during the 2019–2020 season, including the first 23 weeks. However, while we observed an increase in ED visits for patients with fever during the second part of the 2019–2020, we did not find an increase in fever encounters during the first 23 weeks. Since shortness of breath is more likely to be associated with COVID-19, while fever can be associated with either COVID-19, flu, or non-influenza ILI, this finding further supports the notion that the observed increase in CLS during the first 23 weeks of the 2019–2020 was more likely due to be to COVID-19 than to flu or non-influenza ILI. Since ED visits are associated with moderate to severe symptoms, there was likely to have been a larger number of VA patients with COVID-19 infections who had mild or no symptoms and did not require emergency care during this period.

While we observed similar overall patterns in CLS, influenza, and non-influenza ILI, rates for CLS differed among the states for the 2019–2020 season, and TX had somewhat lower rates during the first 23 weeks compared to CA and FL. This could reflect the spread of SARS-CoV-2 from coastal states to the middle of the country. Travel from Asian countries, specifically China, might have played a role in increased CLS rates in CA during the early 2019–2020 because Los Angeles, New York, and San Francisco receive the highest volume of passengers from China in North America [28]. In FL, the early increase in CLS rates could reflect the late fall/early winter movement of residents from New York and other northeastern states [29]. TX might have had fewer travelers coming from regions where SARS-CoV-2 was circulating during this period.

The observed patterns later in the 2019–2020 indicate a substantial increase in CLS, but a decrease in influenza and non-influenza ILI after calendar week 12 of 2020. A study by Wiemken et al., 2020, examining CDC’s FluView Interactive system reported a 76% decrease in influenza-positive diagnoses, but a 27% increase in non-influenza ILI between weeks 9 and 12 of 2020 [30]. Since it did not differentiate between CLS and non-influenza ILI, the study concluded that the observed increases in non-influenza ILI were likely due to undetected COVID-19 cases, which is consistent with our observations of an increase in CLS during the same period. The CLS patterns showed a large spike in mid/late March 2020, and a smaller one during late July/early August 2020 (Figure 1 and Figure S1a). The increase in CLS rates at around week 12 could be due to several factors, including a COVID-19 surge domestically as well as a substantial decrease in the number of non-COVID ED visits. A decrease in ED visits (45–65% overall) was reported due to stay-at-home orders resulting in fewer motor vehicle accidents as well as hesitancy by patients to seek ED services due to a fear of contracting COVID-19 [31,32,33]. A 45% decrease in hospital admissions was observed at the VA between weeks 11 and 16 of 2020 [34]. During March-September 2020, FL showed the highest rates for CLS, while CA and TX had substantially lower CLS rates. This might be due to individuals traveling from the New York region to FL during the NY COVID-19 surge in April–May 2020, as well as the larger number of older residents in FL. However, CLS risks were almost identical in all three states, corresponding to a significant increase in CLS during early 2019–2020 compared to past seasons (Table 2). Risks for non-influenza ILI showed minimal variation across the states and seasons, indicating little change between 2019–2020 and the previous seasons (Table 2). Unlike the previous studies, we modeled CLS, influenza, and non-influenza ILI separately, which allowed us to observe consistent increases in CLS, but not in the rest of ILI, indicating that the increase in ED visits during early 2019–2020 was likely to have been due to COVID-19.

The study has limitations. We examined ED visits among Veterans who used the VA. VA users are older than the US adult population, present with more chronic, physical, and mental conditions, are less educated, have lower incomes, and about 90% are men [19,20]. Given these limitations, the CLS rates in our study might be higher than among the general population [35,36]. We used the VA clinical and administrative data for ED visits that did not include ED visits by VA patients to non-VA facilities. Additionally, because most EDs at the VA do not accept ambulance patient transfers without prior arrangements, many VA ED patients are walk-ins, and therefore might have fewer or milder symptoms. On average, about 15% of patients at non-VA EDs arrive by ambulance, an indicator that increases with age and acuity [37]. Given that VA patients are older and sicker than the general population, the lack of ambulance transport to VA is unlikely to impact our results.

The Veterans who visited VA EDs for CLS, influenza, or non-influenza ILI are most likely to have acquired infections from the communities in which they live. Consequently, our findings suggest COVID-19 spread at the community level in CA, TX, and FL earlier than previously reported. Additionally, we relied on ICD-10-CM diagnoses, some but not all of which indicated a confirmed influenza virus. Similarly, we did not use laboratory tests to confirm non-influenza ILI diagnoses. However, it is unlikely that the use of ICD-10-CM during the 2019–2020 season differed from previous seasons, and therefore this should not have affected the observed differences between 2019–2020 and the other seasons. Furthermore, it was not possible to use tests to confirm the presence of COVID-19 at the beginning of the 2019–2020 season. Instead, we relied on the case definition to identify patients with CLS. The observed CLS patterns for VA users in late 2019–2020 were consistent with the patterns of COVID-19 spread in CA, TX, and FL, allowing us to conclude that the increases we observed in CLS early in 2019–2020 indicate the likely presence of COVID-19 early in 2019–2020 in these states.

## 5. Conclusions

Tracking ED encounters suggests that SARS-CoV-2 may have been circulating in CA, TX, and FL earlier than was originally considered, and perhaps even before COVID-19 cases were identified in the US. Our study adds to the growing body of new evidence that COVID-19 was present in the US much earlier than originally detected [9,10,11]. Using the VA CDW database, we found statistically significant increases in risk for CLS during the 2019–2020 season, including during its first 23 weeks, in CA, FL, and TX. Separating CLS from other ILI allowed us to observe the increase only in trends indicative of COVID-19.

VA administrative and clinical data are reported by VA providers in real time, and could, in hindsight, have suggested the possibility of an unknown illness circulating in the communities. Similar real-time studies in the future have the potential to be an early warning for other infectious diseases. Such effforts could potentially supplement weekly ILI outpatient surveillance system [38]. Therefore, tracking ILI through VA administrative and clinical data could potentially improve syndromic surveillance by serving as a supplementary tool to identify emerging infectious threats before they lead to high rates of hospitalizations and deaths.

## Figures and Tables

**Table 1:** Number of ED visits, rate per 1000 ED encounters during first 23 weeks of five flu seasons (1 October–29 February), and difference in rates between each of the seasons and the 2019–2020 flu season for CLS, influenza, and non-influenza ILI by state.

Season	Number of Visits	Rate per 1000	Percentage Change in Rates Comparing 2019–2020 to Previous Seasons	Number of Visits	Rate per 1000	Percentage Change in Rates Comparing 2019–2020 to Previous Seasons	Number of Visits	Rate per 1000	Percentage Change in Rates Comparing 2019–2020 to Previous Seasons
**COVID-like symptoms**	**California**			**Texas**			**Florida**		
2015–2016	4134	45	−29% ***	3209	50	−23% ***	3569	48	−26% ***
2016–2017	4970	51	−19% **	3525	51	−22% ***	4421	53	−18% **
2017–2018	5058	51	−19% **	4176	60	−8% **	4370	52	−20% ***
2018–2019	4838	51	−19% **	4219	62	−5% *	4340	53	−18% **
4-season Average	4750	49	−22% **	3782	56	−14% ***	4175	52	−20% ***
2019–2020 *	5388	63		4132	65		4956	65	
**Influenza**	**California**			**Texas**			**Florida**		
2015–2016	196	2	−71% **	102	2	−82% ***	162	2	−71% ***
2016–2017	308	3	−57% **	242	3	−73% ***	254	3	−57% ***
2017–2018	973	10	43%	1185	17	55%	944	11	57%
2018–2019	216	2	−71% **	243	4	−64% **	275	3	−57% **
4-season average	423	4	−43% *	443	6	−65% *	411	5	−29%
2019–2020	596	7		683	11		541	7	
**Non-influenza ILI**	**California**			**Texas**			**Florida**		
2015–2016	7528	81	7%	5243	81	−9%	5799	78	−7%
2016–2017	8004	81	7%	5923	85	−4%	7658	92	10%
2017–2018	8696	88	16%	7294	105	18% *	7909	94	12%
2018–2019	6684	71	−7%	6187	92	3%	7066	86	2%
4-season average	7728	80	5%	6162	91	2%	7108	88	5%
2019–2020	6528	76		5641	89		6478	84	

* *p* < 0.05; ** *p* < 0.001; *** *p* < 0.0001.

**Table 2:** Adjusted Risk Ratios between 23 weeks (1 October–29 February 29) of 2019–2020 flu season compared to each of four previous seasons for ED visits among VA patients with CLS, influenza, and non-influenza ILI by state.

Season (Ref 2019–2020)	Adjusted Risk Ratio	Adjusted Risk Ratio	Adjusted Risk Ratio
	**CA COVID-Like Symptoms**	**TX COVID-Like Symptoms**	**FL COVID-Like Symptoms**
2015–2016	0.72 *** (0.69–0.75)	0.75 *** (0.72–0.79)	0.72 *** (0.69–0.75)
2016–2017	0.81 *** (0.78–0.85)	0.77 *** (0.73–0.81)	0.79 *** (0.76–0.82)
2017–2018	0.80 *** (0.77–0.84)	0.89 *** (0.85–0.93)	0.83 *** (0.80–0.87)
2018–2019	0.82 *** (0.79–0.86)	0.93 *** (0.89–0.97)	0.81 *** (0.78–0.84)
**Season (Ref 2019–2020)**	**CA Influenza diagnoses**	**TX Influenza diagnoses**	**FL Influenza diagnoses**
2015–2016	0.29 *** (0.24–0.34)	0.15 *** (012–0.19)	0.35 *** (0.30–0.42)
2016–2017	0.45 *** (0.39–0.52)	0.32 *** (0.27–0.37)	0.43 ** (0.37–0.50)
2017–2018	1.24 ** (1.11–1.38)	1.46 *** (1.32–1.62)	1.51 ** (1.36–1.68)
2018–2019	0.34 *** (0.29–0.40)	0.35 *** (0.30–0.40)	0.50 *** (0.43–0.57)
**Season (Ref 2019–2020)**	**CA non-influenza ILI diagnoses**	**TX non-influenza ILI diagnoses**	**FL non-influenza ILI diagnoses**
2015–2016	1.04 * (1.00–1.08)	0.93 *** (0.90–0.97)	0.92 *** (0.89–0.96)
2016–2017	1.05 * (1.01–1.09)	1.10 (1.06–1.17)	1.07 *** (1.04–1.11)
2017–2018	1.08 *** (0.004–0.009)	1.10 *** (1.06–1.14)	1.06 ** (1.02–1.09)
2018–2019	0.91 *** (0.88–0.94)	1.01 (0.97–1.05)	0.99 (0.96–1.02)

* *p* < 0.05; ** *p* < 0. 001; *** *p* < 0.0001.

## Data Availability

De-identified, aggregated data can be provided upon request.

## Supplementary Materials

The following supporting information can be downloaded at: https://www.mdpi.com/article/10.3390/v14020200/s1, Figure S1: a–c: Rates per 1000 ED encounters for COVID-like symptoms, influenza diagnoses, and non-influenza ILI by state (CA, TX, FL) and by flu season (2015–2020)., Figure S2: a–c: Rates per 1000 ED encounters for shortness of breath, cough, and fever (CA, TX, FL) (2015–2020), Table S1: Demographic characteristics of VA patients visiting ED from 1 October 2015–30 September 2020 by state and season. Table S2: Demographic characteristics of VA patients visiting ED for CLS from 1 October 2015–30 September 2020 by state and season.
