# Supplementary material for: Using an Administrative and Clinical Database to Determine the Early Spread of COVID-19 at the US Department of Veterans Affairs during the Beginning of the 2019–2020 Flu Season: A Retrospective Longitudinal Study
Source: Viruses. 2022 Jan 20;14(2):200. doi: 10.3390/v14020200 (PMC8879908; doi:10.3390/v14020200)
Supplement: Supplementary file 1 [file viruses-14-00200-s001.zip › viruses-1530778-supplementary.pdf]

Online Supplement

Figures S1 a-c. Rates per 1,000 ED encounters for COVID-like symptoms, influenza diagnoses, and non-influenza ILI by state (CA, TX, FL) and by flu season (2015-2020).

Figures S2 a-c. Rates per 1,000 ED encounters for shortness of breath, cough, and fever (CA, TX, FL) (2015-2020).

Table S1. Demographic characteristics of VA patients visiting ED from October 1, 2015 – September 30, 2020 by state and season.

Table S2. Demographic characteristics of VA patients visiting ED for CLS from October 1, 2015 – September 30, 2020 by state and season.

Figures S1 a-e. Rates per 1,000 ED encounters for COVID-like symptoms, influenza diagnoses, and non-influenza ILI by state (CA, TX, FL) and by flu season (2015-2020).

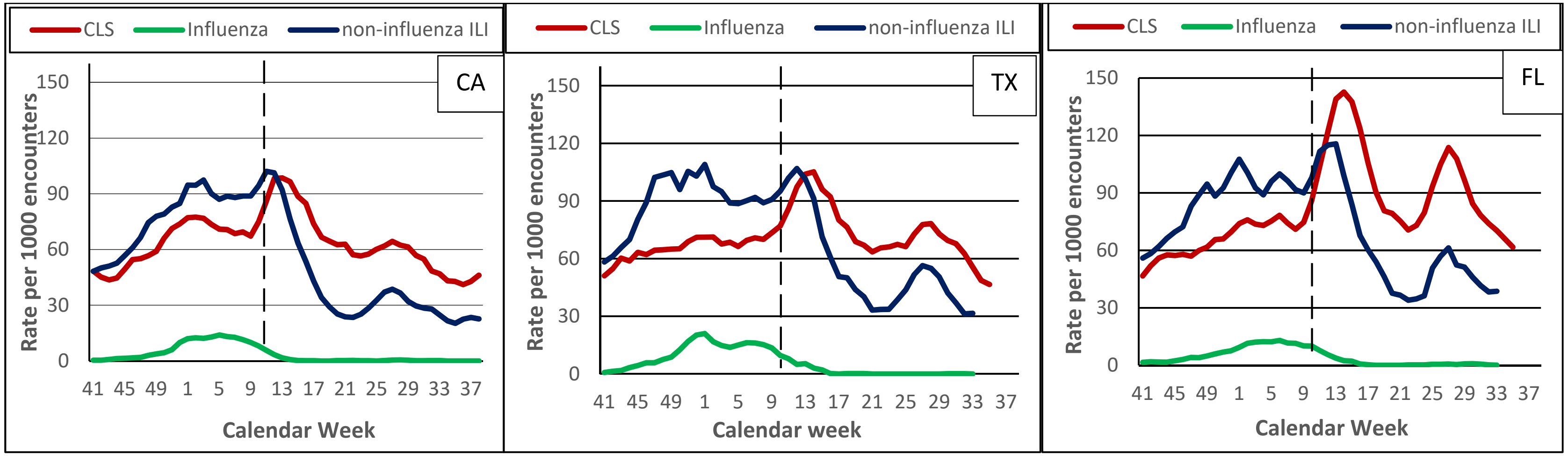

Figure S1 a. 2019-2020

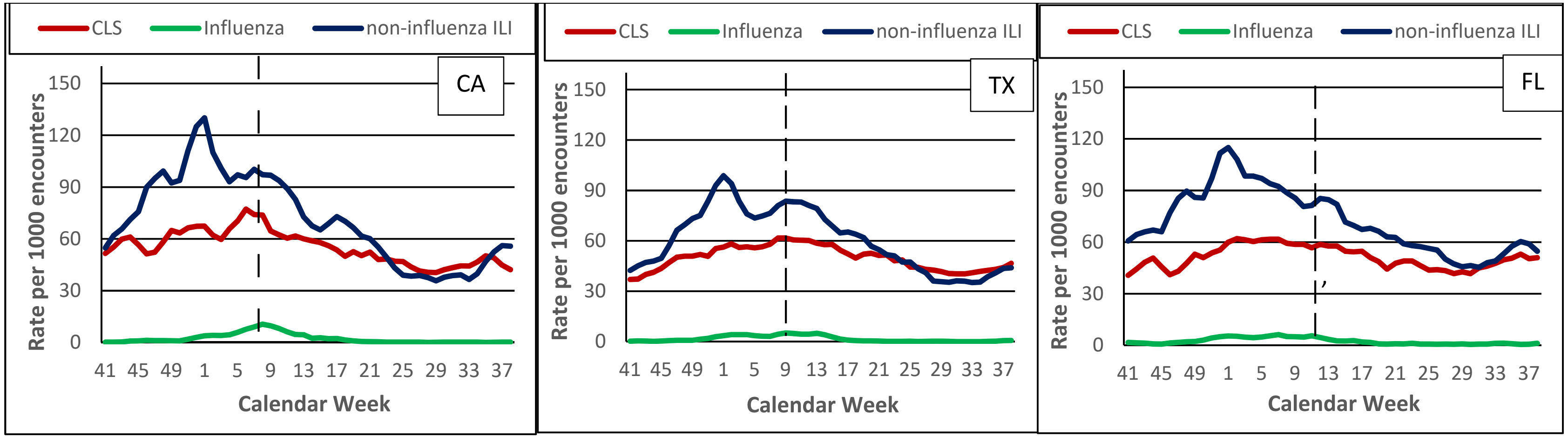

Figure S1 b. 2018-2019

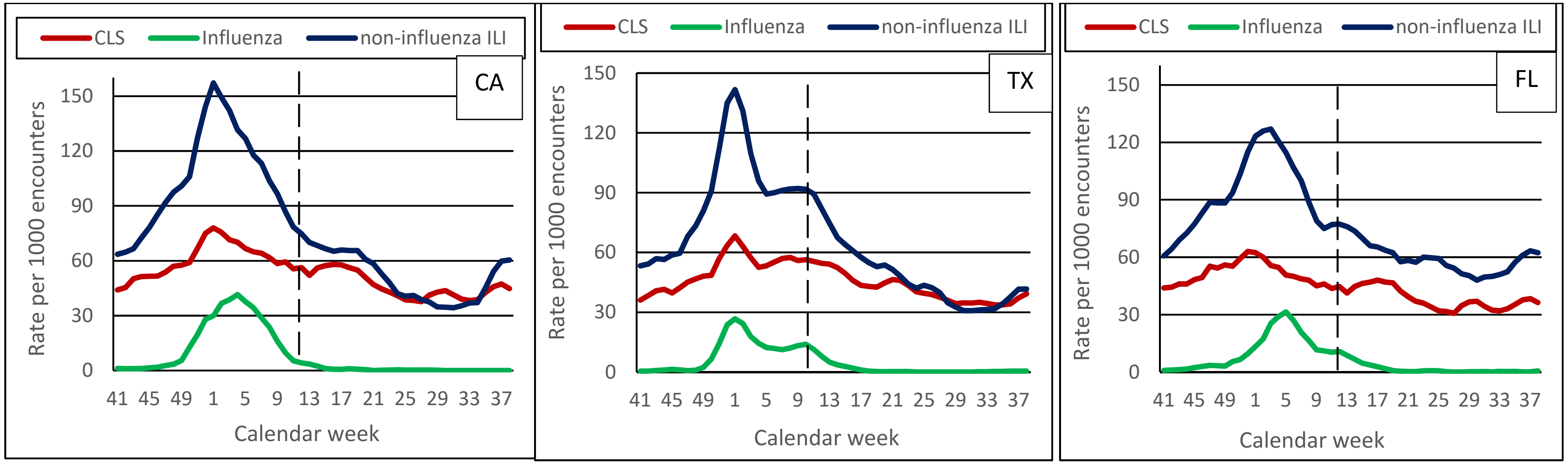

Figure S1 c. 2017-2018

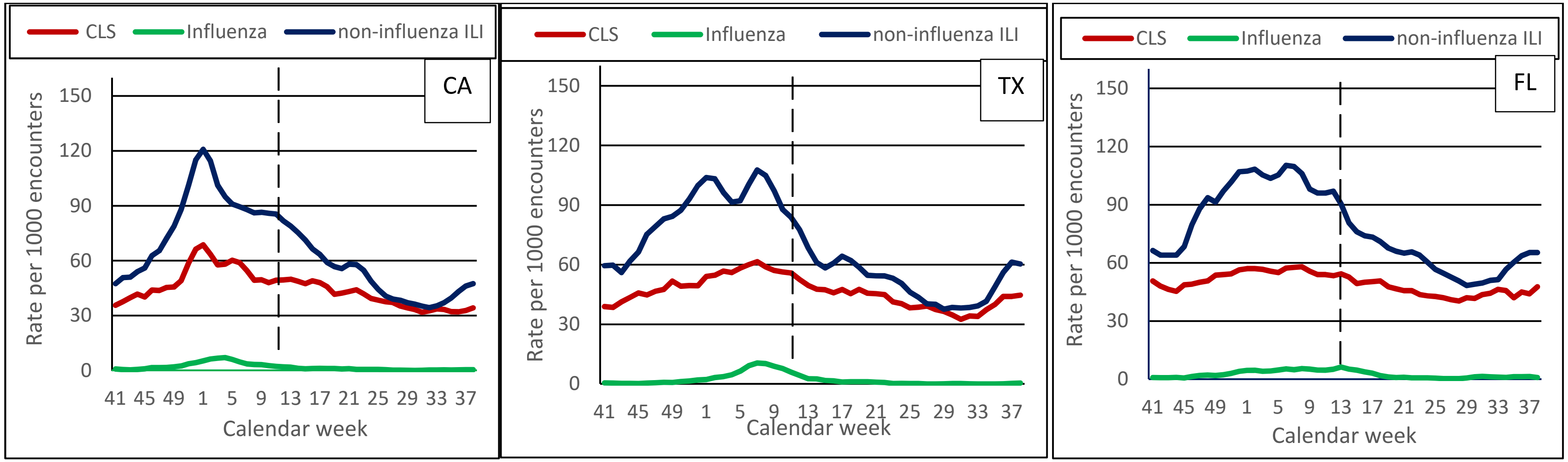

Figure S1 d. 2016-2017

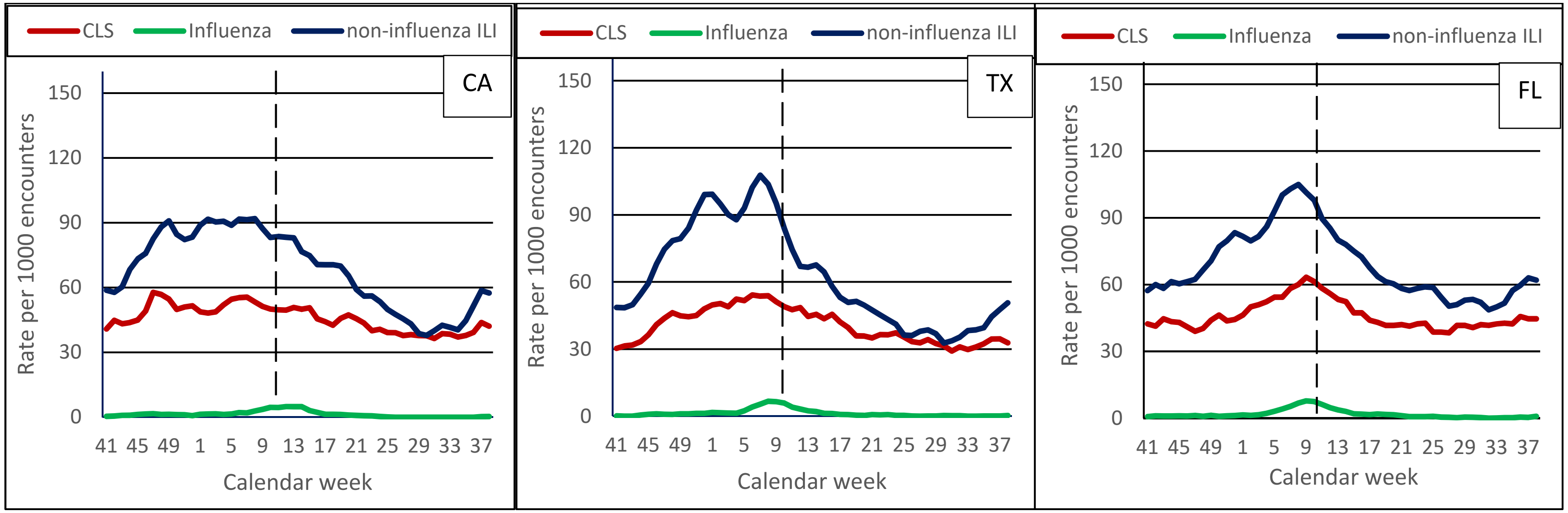

Figure S1 e. 2015-2016

Figures S2 a-c. Rates per 1,000 ED encounters for shortness of breath, cough, and fever (CA, TX, FL) (2015-2020).

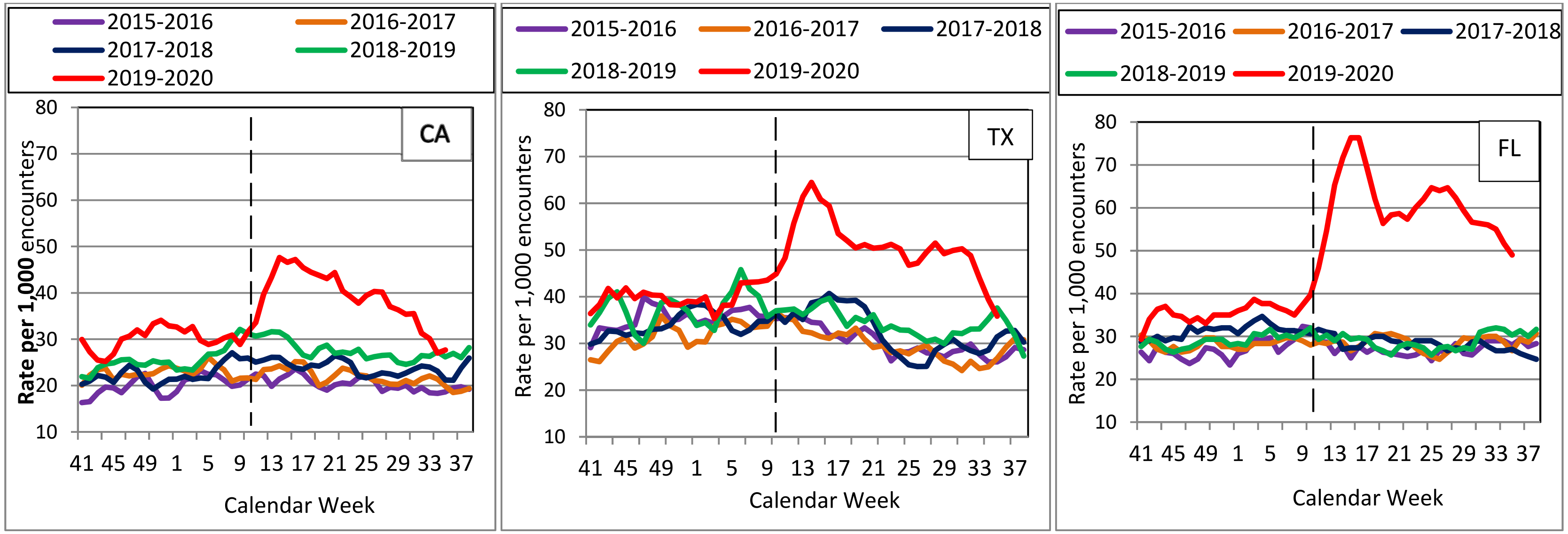

Figure S2 a. Shortness of breath

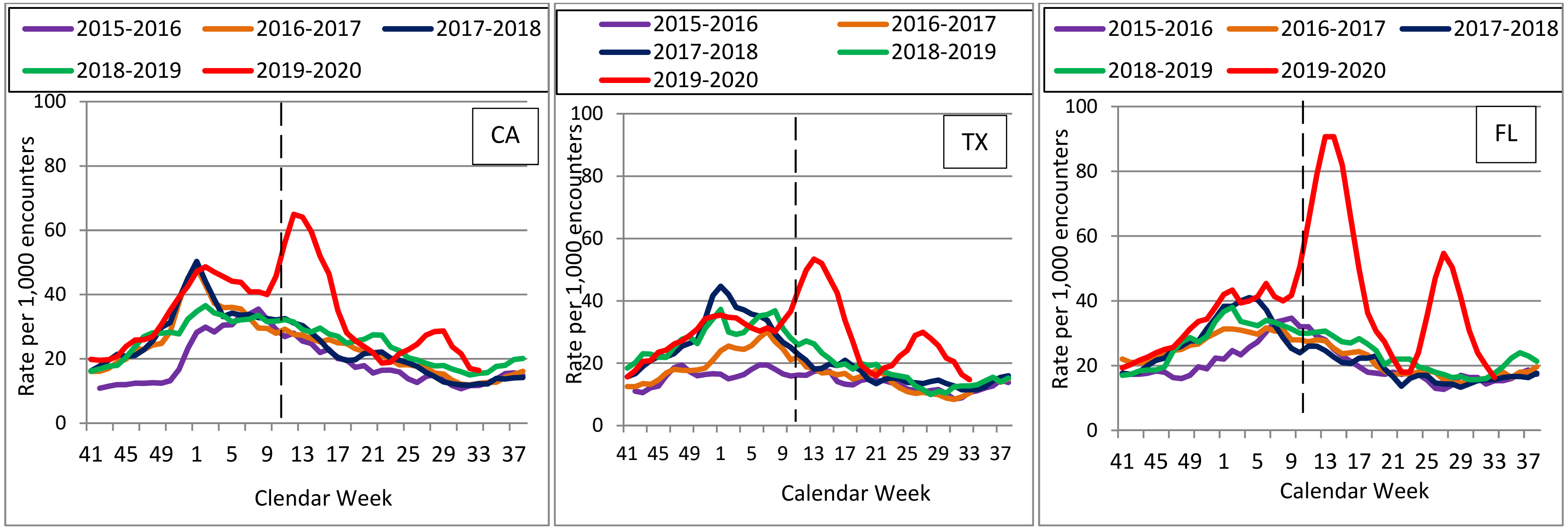

Figure S2 b. cough

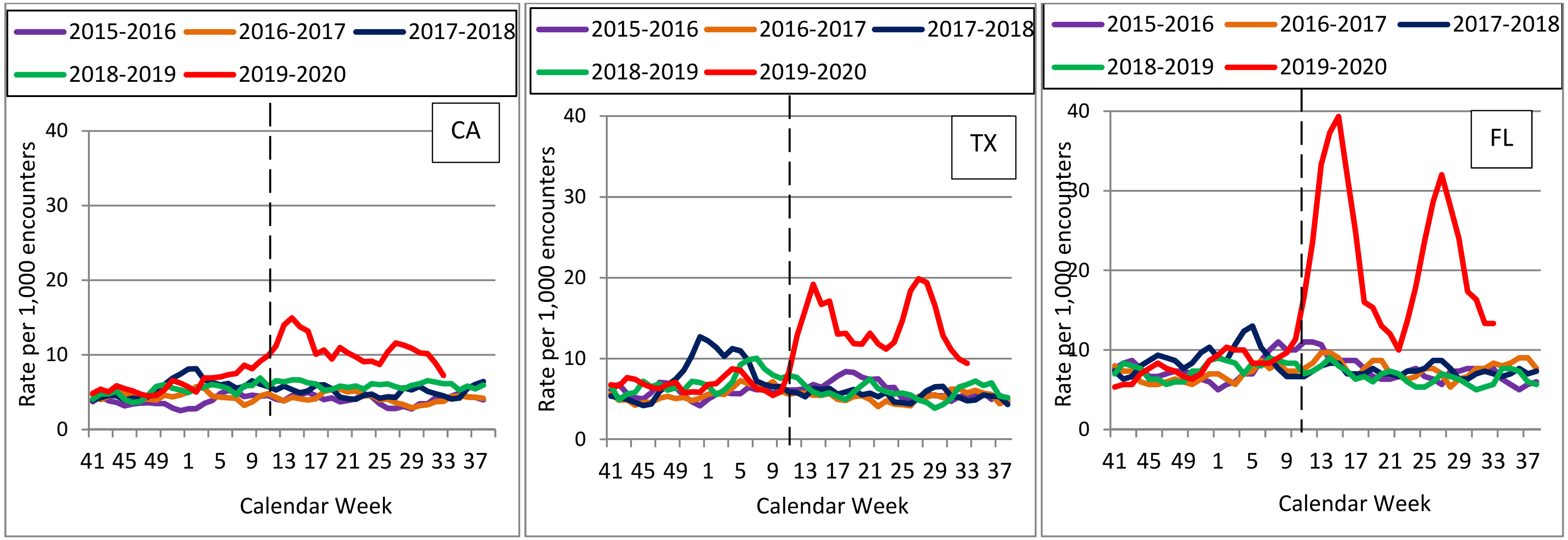

Figure S2 c. fever

Table S1. Demographic characteristics of VA patients visiting ED from October 1, 2015 – September 30, 2020 by state and season.

All ED visits	2015-2016	2016-2017	2017-2018	2018-2019	2019-2020
**Number of patients***					
California	107,998 (21%)	111,611 (21%)	110,850 (21%)	103,842 (20%)	85,725 (17%)
Texas	80,979 (21%)	84,030 (21%)	82,382 (21%)	79,512 (20%)	65,541 (17%)
Florida	94,180 (20%)	98,141 (21%)	98,357 (21%)	97,491 (21%)	79,459 (17%)
**Gender Male (%)**					
California	97,118 (90%)	100,139 (90%)	99,243 (90 %)	93,194 (90%)	76,962 (90%)
Texas	69,719 (86%)	72,085 (86%)	70,136 (85%)	67,630 (85%)	55,728 (85%)
Florida	83,515 (89%)	86,703 (88%)	86,789 (88%)	85,831 (88%)	69,901 (88%)
**Marital status Married**					
California	40,117 (37%)	41,520 (37%)	41,335 (38%)	39,255 (39%)	32,322 (38%)
Texas	42,278 (45%)	44,670 (46%)	45,397 (46%)	45,024 (46%)	36,748 (46%)
Florida	42,278 (45%)	44,670 (46%)	45,397 (46%)	45,024 (46%)	36,748 (46%)
**Divorced/Separated/ Widowed**					
California	42,279 (39%)	42,652 (38%)	41,666 (38%)	38864 (37%)	31,650 (37%)
Texas	32,114 (40%)	33,067 (40%)	31,494 (38%)	30,102 (38%)	24,736 (38%)
Florida	35,713 (38%)	36,378 (37%)	35,863 (36%)	34856 (36%)	28,157 (35%)
**Single/Never Married**					
California	24,725 (23%)	26,196 (23%)	26,378 (23%)	24,580 (24%)	20,444 (24%)
Texas	12,316 (15%)	12,816 (15%)	12,887 (16%)	12,592 (16%)	10,212 (16%)
Florida	15,608 (17%)	16,339 (17%)	16,326 (17%)	16,770 (17%)	13,833 (17%)
**Unknown/Missing**					
California	876 (1%)	1,242 (1%)	1,469 (1%)	1,143 (1%)	1309 (1%)
Texas	633 (1%)	747 (1%)	696 (1%)	627 (1%)	620 (1%)
Florida	581 (0.6%)	754 (0.8%)	771 (0.8%)	841 (0.9%)	721 (0.9%)
**Race: White**					
California	57,667 (53%)	58,596 (53%)	57,603 (52%)	53,615 (52%)	43,043 (50%)
Texas	38,422 (48%)	39,518 (47%)	37,791 (46%)	35,767 (45%)	28,687 (44%)
Florida	60,028 (63%)	61,620 (63%)	60,040 (61%)	58,986 (61%)	47,232 (60%)
**African American**					
California	19,841 (18%)	20,781 (19%)	20,665 (19%)	19,839 (19%)	16,491 (19%)
Texas	24,134 (30%)	25,415 (30%)	25,832 (32%)	25,465 (32%)	21,385 (33%)
Florida	18,989 (20%)	19,594 (20%)	20,093 (20%)	20,361 (21%)	17,086 (22%)
**Latino**					
California	14,418 (14%)	15,013 (13%)	15,016 (14%)	14,557 (14%)	12,546 (15%)
Texas	10,802 (13%)	11,231 (13%)	11,305 (14%)	11,123 (14%)	9,437 (14%)

All ED visits	2015-2016	2016-2017	2017-2018	2018-2019	2019-2020
Florida	8,597 (9%)	10,005 (10%)	11,176 (11%)	11,155 (11%)	9,205 (12%)
**Asian**					
California	4,069 (2%)	4,265 (4%)	4,461 (4%)	4,278 (4%)	3,595 (4%)
Texas	448 (0.5%)	497 (1%)	515 (1%)	507 (1%)	436 (1%)
Florida	318 (0.3%)	331 (0.3%)	407 (0.4%)	410 (0.4%)	385 (0.5%)
**Native American/Hawaiian**					
California	3,025 (3%)	3,164 (3%)	3,245 (3%)	3,072 (3%)	2,600 (3%)
Texas	1,399 (2%)	1,496 (2%)	1,472 (2%)	1,446 (2%)	1,204 (2%)
Florida	1,274 (1%)	1,410 (1%)	1,459 (1%)	1,415 (1%)	1,206 (1.5%)
**Unknown/Missing**					
California	8,978 (8%)	9,791 (9%)	9,855 (9%)	8481 (8%)	7,450 (9%)
Texas	5,774 (7%)	58,73 (7%)	5467 (7%)	5,204 (7%)	4392 (7%)
Florida	4,974 (5%)	5,181 (5%)	51,83 (5%)	5,164 (5%)	4,345 (5%)
**Mean age**					
California	58	59	59	60	60
Texas	56	56	57	57	58
Florida	60	60	60	60	61
**Number of ED visits per patient per year**					
California	2.0	2.0	2.0	2.0	2.0
Texas	1.9	1.9	1.9	1.9	1.9
Florida	1.9	1.9	2	2	1.9
**Number of weeks visited per year**					
California	1.9	1.9	1.9	1.9	1.8
Texas	1.8	1.8	1.8	1.8	1.8
Florida	1.8	1.9	1.8	1.9	1.8

* Percent of 5-year total, the rest of percentages are from the season total.

Table S2. Demographic characteristics of VA patients visiting ED for CLS from October 1, 2015 – September 30, 2020 by state and season.

COVID-like symptoms	2015-2016	2016-2017	2017-2018	2018-2019	2019-2020
**Number of patients***					
California	7,426 (7%)	8,476 (8%)	8,668 (8%)	8,888 (8.5%)	8,974 (10%)
Texas	5,809 (7%)	6,223 (7%)	6,948 (8%)	7,096 (9%)	6,937 (11%)
Florida	7,252 (7%)	8,200 (8%)	8,248 (8%)	8439 (9%)	10,082 (13%)
**Gender Male (%)**					
California	6,684 (90%)	7,650 (90%)	7,831 (90%)	8,058 (91%)	8,081 (90%)
Texas	5,163 (88%)	5,501 (88%)	6,070 (87%)	6,239 (88%)	5,992 (86%)
Florida	6471 (89%)	7,362 (90%)	7,418 (90%)	7,587 (90%)	8,941 (89%)
**Marital status Married**					
California	3,053 (41%)	3,484 (41%)	3,542 (41%)	3,575 (40%)	3,524 (39%)
Texas	2,765 (48%)	2,951 (47%)	3,266 (47%)	3,245 (46%)	3,317 (48%)
Florida	3,624 (50%)	4,079 (50%)	4,135 (50%)	4,313 (51%)	4,907 (49%)
**Divorced/Separated/ Widowed**					
California	2,943 (40%)	3,278 (39%)	3, 326 (39%)	3,447 (39%)	3,339 (37%)
Texas	2,386 (41%)	2518 (41%)	2805 (41%)	2901 (41%)	2665 (38%)
Florida	2,727 (37%)	3,068 (37%)	3,044 (37%)	2,992 (35%)	3630 (36%)
**Single/Never Married**					
California	1,400 (19%)	1,655 (20%)	1,750 (20%)	1,815 (20%)	2,014 (22%)
Texas	641 (11%)	727 (12%)	849 (12%)	927 (13%)	917 (13%)
Florida	879 (12%)	1,023 (12%)	1,033 (13%)	1,103 (13%)	1,497 (15%)
**Unknown/Missing**					
California	30 (0.4%)	59 (0.7%)	50 (0.6%)	51 (0.6%)	97 (1%)
Texas	17 (0.3%)	27 (0.4%)	26 (0.4%)	23 (0.3%)	38 (1%)
Florida	22 (0.3%)	30 (0.3%)	26 (0.3%)	31 (0.4%)	48 (0.5%)
**Race: White**					
California	4,002 (54%)	4,575 (54%)	4,594 (53%)	4,695 (53%)	4,469 (50%)
Texas	2,963 (51%)	3,127 (50%)	3,342 (48%)	3, 330 (47%)	3,101 (45%)
Florida	4,841 (67%)	5,367 (65%)	5,351 (65%)	5,425 (64%)	6,116 (61%)
**African American**					
California	1,495 (20%)	1,662 (20%)	1,670 (19%)	1,794 (20%)	1,745 (19%)
Texas	1,869 (32%)	1,937 (31%)	2093 (30%)	2,295 (32%)	2,312 (33%)
Florida	1,356 (19%)	1,523 (19%)	1,546 (19%)	1,681 (20%)	2,150 (21%)
**Latino**					
California	863 (12%)	958 (11%)	1,011 (12%)	1,039 (12%)	1,274 (14%)
Texas	570 (10%)	643 (10%)	907 (13%)	872 (12%)	933 (13%)

COVID-like symptoms	2015-2016	2016-2017	2017-2018	2018-2019	2019-2020
Florida	585 (8%)	792 (10%)	846 (10%)	794 (9%)	1,147 (11%)
**Asian**					
California	314 (4%)	385 (5%)	415 (5%)	396 (4%)	439 (5%)
Texas	18 (0.3%)	32 (0.5%)	38 (0.5%)	37 (0.5%)	36 (0.5%)
Florida	23 (0.3%)	19 (0.2%)	21 (0.3%)	24 (0.3%)	45 (0.5%)
**Native American/Hawaiian**					
California	216 (3%)	227 (3%)	270 (3%)	249 (3%)	277 (3%)
Texas	77 (1%)	110 (2%)	115 (2%)	138 (2%)	148 (2%)
Florida	92 (1%)	121 (1%)	99 (1%)	108 (1%)	145 (1%)
**Unknow/Missing**					
California	536 (7%)	669 (8%)	708 (8%)	715 (8%)	770 (9%)
Texas	312 (5%)	374 (6%)	453 (7%)	424 (6%)	407 (6%)
Florida	355 (5%)	378 (5%)	385 (5%)	407 (5%)	479 (5%)
**Mean age**					
California	63	63	64	64	63
Texas	63	63	62	63	62
Florida	63	63	62	63	62
**Number of visits per patient per year**					
California	1.2	1.2	1.2	1.2	1.2
Texas	1.2	1.2	1.2	1.2	1.2
Florida	1.2	1.2	1.2	1.2	1.2

* % are from season total listed in Table1; all the other percentages are from COVID-like symptoms season total.
